# The Major Role of Type 2 Inflammation in Asthma: From the Perspective of Immunological Mechanism

**DOI:** 10.1155/bmri/5663596

**Published:** 2026-01-20

**Authors:** Lanying Cheng, Lihong Sun

**Affiliations:** ^1^ Department of Pediatric Pulmonology, The First Affiliated Hospital of Guangzhou Medical University, State Key Laboratory of Respiratory Disease, Guangzhou, China, gzhmc.edu.cn

**Keywords:** asthma, epithelial cell, immune cells, mechanism, Type 2 inflammation

## Abstract

Asthma is a heterogeneous disease that affects individuals of diverse age groups globally and exhibits variable responses to different treatments. Type 2 inflammation contributes to the pathogenesis of asthma through the production of cytokines IL‐4, IL‐5, and IL‐13, thereby inducing characteristic features of asthma such as elevated eosinophil levels and airway hyperresponsiveness. The findings of the current study indicate that over 50% of individuals suffering from asthma exhibit Type 2 inflammation, with a higher prevalence observed in severe asthma. Moreover, biologics specifically designed to target Type 2 inflammation not only demonstrate favorable outcomes in treating severe asthma but also offer promising prospects for managing this condition. Therefore, it is of paramount importance to comprehend the intricate mechanisms of Type 2 inflammation in asthma. This article is aimed at providing an overview of the involvement of structural cells, innate immune cells, and adaptive immune cells in relation to Type 2 inflammation in asthma.

## 1. Introduction

Asthma is a prevalent and heterogeneous chronic respiratory disorder affecting 1%–29% of the global population, characterized by persistent airway inflammation and hyperresponsiveness, leading to respiratory symptoms including wheezing, cough, and chest tightness. The airflow restriction in most asthma patients is reversible and can be resolved spontaneously or improved with medication [1]. However, individuals with chronic persistent asthma may result in airway remodeling due to changes in airway structure cells, leading to persistent or incomplete reversibility of airflow restriction. Due to the heterogeneity of asthma, different clinical manifestations and severity of the disease result in distinct responses to treatment. With extensive investigation into the molecular mechanisms underlying asthma, we have classified it into two subtypes based on immune response: “T2 high” and “T2 low” [2]. William et al. [3] pointed out that Type 2 inflammation–related asthma accounts for approximately 50%–70% of cases. However, due to the significant influence of ICS or OCS usage on Type 2 inflammation indicators, some patients may be misclassified as having a “low T2” phenotype. Consequently, it is plausible that the prevalence of “high T2” asthma patients may be even higher. Asthma accompanied by Type 2 inflammation primarily involves the stimulation of Th2 cells and Type 2 innate lymphoid cells (ILC2) in response to allergens and viruses, leading to the secretion of Type 2 cytokines including IL‐4, IL‐13, and IL‐5 (interleukin‐5). These cytokines subsequently induce an upregulation of biomarkers associated with Type 2 inflammation such as IgE and eosinophils, ultimately resulting in airway inflammation. The emergence of targeted biologic therapy for Type 2 inflammation has marked a significant breakthrough in the uncontrolled persistent asthma [4], and comprehending the pivotal role played by type 2 inflammation in asthma pathogenesis can pave the way for further advancements in personalized treatment strategies.

Type 2 immune response is initiated when the airway is stimulated or damaged by allergens, pathogens, etc., through intricate interactions among innate and adaptive immune cells along with structural cells. Specifically, upon stimulation, these cells secrete Type 2 cytokines (IL‐4, IL‐5, IL‐9, and IL‐13) [5] which promote Type 2 inflammatory response in the body and lead to characteristic symptoms of asthma and airway remodeling.

**Figure 1:**
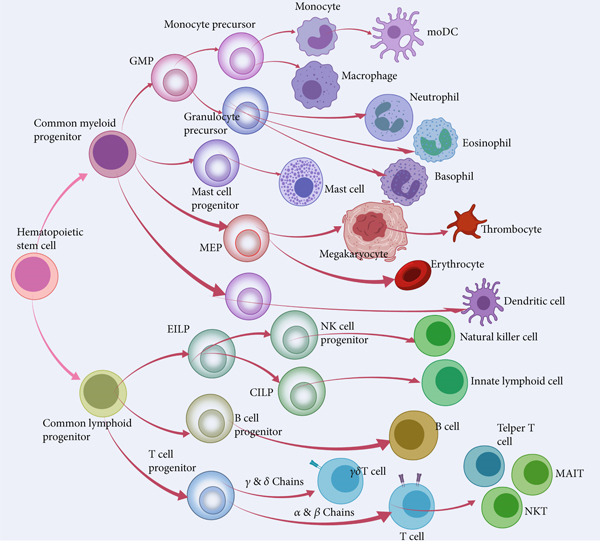
The development of various cells in the framework of hematopoiesis. GMP, granulocyte macrophage progenitor; MEP, megakaryocyte erythroid progenitor; moDC, monocyte‐derived dendritic cell.

Currently, the development of biologics targeting specific molecular pathways in asthma pathogenesis has emerged as a prominent research focus for personalized treatment and addressing refractory asthma [6]. This review will primarily focus on the immunological basis of Type 2 inflammation–induced asthma and comprehensively explore the intricate involvement of various types of cells (including immune cells and structural cells, whose cell sources are shown in Figure 1) in the pathogenesis of asthma, spanning from animal models to clinical implications.

## 2. Structural Cells Associated With Type 2 Inflammation in Asthma

### 2.1. Epithelial Cells and the Cytokines They Secrete

#### 2.1.1. The Epithelial Barrier Is the First Line of Defense

In recent years, an increasing body of research has demonstrated the crucial role of airway epithelial cells in the pathogenesis of asthma. Airway epithelial cells not only serve as the primary physical barrier against the external environment but also play a pivotal role in orchestrating immune responses. The main cellular components of the airway epithelium include ciliated epithelial cells, goblet cells, airway basal cells, and club/clara cells, [7] as well as rare but specialized neuroendocrine cells, isolated chemosensory cells, and ionic cells [8, 9]. The junction between these airway epithelial cells forms an apical junction complex composed of tight junctions and adhesive junctions, which establishes a physical barrier to safeguard the body [10]. The disruption of the epithelial barrier represents one of the initial events that precipitates the commencement of asthma. IgE crosslinking with Fc*ε*RI leads to the downregulation of claudin‐18 and E‐cadherin expression, thereby disrupting the tight junctions between epithelial cells. Compared to non‐Type 2 inflammatory asthma patients, severe asthma patients with Type 2 inflammation demonstrate a significant reduction in the expression of claudin‐18 and E‐cadherin in bronchial biopsies. Moreover, inhibition of eosinophilic inflammation can also prevent loss of E‐cadherin [11–13]. Allergenic proteins derived from pollen, house dust mites, and fungi exhibit protease activity and possess the ability to cleave tight junctions, thereby disrupting the integrity of the epithelial barrier. Inhaled allergens have been demonstrated to augment the expression of CYP27A1 by inducing 27‐HC, thereby disrupting the integrity of tight junctions [14]. In murine models, fungal proteases directly disrupt adhesion junctions, thereby inducing a surge of calcium influx and initiating an allergic inflammatory response [15]. When allergens assail epithelial cells, they disrupt their barrier function, initiating an allergic inflammatory response and facilitating the onset and progression of asthma.

#### 2.1.2. Epithelial‐Derived Cytokines Serve as the Trigger

**Figure 2:**
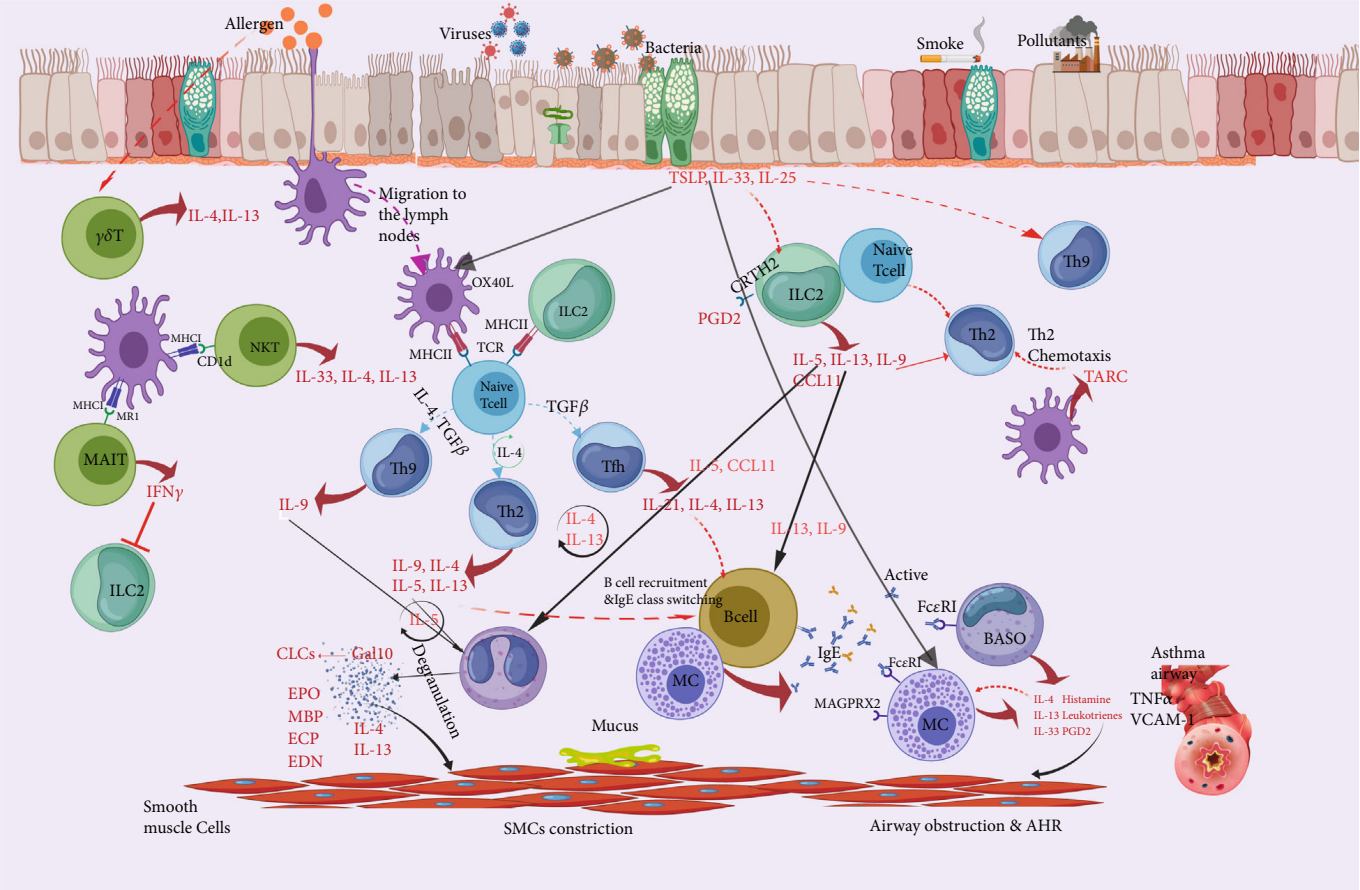
Immunological mechanisms underlying Type 2 inflammation in asthma.

Epithelial cells not only constitute a physical barrier defense but also produce cytokines that act upstream of Type 2 immune responses. Exposure to allergens, viruses, or bacteria triggers the pattern recognition receptor (PRR) on epithelial cells, leading to the induction of alarm hormones (such as IL‐25, IL‐33, and TSLP) and chemokines (including CCL2 and CCL20), subsequently initiating a cascade that activates downstream immune cells (including dendritic cells [DCs], ILC2s, and Th2 cells), thereby promoting Type 2 immune inflammatory response (Figure 2). Numerous studies have demonstrated the crucial role of epithelial‐derived alarmins in Type 2 immune response; however, the precise assessment of each individual alarmin remains challenging.

TSLP is a pleiotropic cytokine, initially identified as a B cell growth factor [16]. Its receptor is a heterodimer consisting of the alpha chain of IL‐7 receptor and the gamma chain of TSLP receptor. In humans, there are two functionally distinct subtypes of TSLP: the short isoform (sfTSLP) associated with promoting homeostasis and the long isoform (lfTSLP) linked to allergic inflammation [17]. Studies conducted in murine models have elucidated the intricate interplay between lfTSLP and immune cells, including ILC2 and DCs expressing TSLPR. This interplay induces the production of IL‐4, IL‐5, and IL‐13, initiating Type 2 inflammation [18]. The findings regarding TSLP and human asthma align with the mouse model, as TSLP promotes the proliferation of CD4+ T cells and enhances Type 2 cytokine production [19]. Additionally, IL‐4 can positively regulate epithelium‐derived TSLP expression [20], thus establishing a positive feedback loop that exacerbates Type 2 inflammation. Interestingly, Louise′s study revealed that the involvement of TSLP in AHR among asthma patients is independent of eosinophilic inflammation, implying its participation in non‐T2 inflammation as well [21]. These findings suggest that TSLP may potentiate the progression of allergic asthma primarily by augmenting the T2 immune response. The anti‐TSLP monoclonal antibody tezepelumab has demonstrated remarkable efficacy in Phase I and II clinical trials, exhibiting improvements in pulmonary function, reduction of eosinophils, and alleviation of asthma symptoms [22]. In addition to treating severe asthma, ecleralimab, a newly developed inhaled anti‐TSLP biologic, holds potential for application in patients with mild atopic asthma [23].

IL‐33, a member of the IL‐1 cytokine family, binds to a membrane‐bound heteroreceptor composed of the functional receptor ST2 and IL‐1RAP. After epithelial injury, allergens stimulate the production of IL‐33 in epithelial cells through the damage‐associated molecular patterns (DAMPs). Subsequently, they bind to ST2 and IL‐1RAP, activating downstream immune cells such as ILC2s, mast cells (MCs), and eosinophils, thereby inducing Type 2 inflammation [24]. The release of IL‐33 is considered to occur early after exposure to allergens, and its levels are significantly increased in bronchial epithelial cells, airway smooth muscle, bronchoalveolar lavage fluid (BALF), and peripheral blood circulation of patients with asthma, showing a correlation with the severity of asthma [25–27]. IL‐33 promotes Type 2 inflammation by exerting its effects on a variety of cells, with particular emphasis on ILC2 cells as the primary targets. Sun et al. [28] revealed that IL‐33 triggers and activates ILC2 within the pulmonary milieu via the PI3K/AKT signaling pathway. Notably, Zhu et al. [29] discovered that in both murine models and asthmatic patients, pyridoxal phosphate (PLP), an active form of vitamin B6, attenuated MDM2‐mediated IL‐33 polyubiquitination and reduced IL‐33 levels via the proteasome pathway, ultimately ameliorating the Type 2 immune response. The clinical trial findings of itepekimab, an anti‐IL‐33 monoclonal antibody, currently demonstrate a significant reduction in blood eosinophil levels and improvement in lung function among patients with moderate to severe asthma [30]. However, a larger Phase 3 clinical trial is warranted to further elucidate its indications.

IL‐25, also known as IL‐17E, belongs to the IL‐17 family. The receptor for IL‐25 is composed of two subunits, namely, IL‐17RA and IL‐17RB [31]. Subsequently, a complex is formed by the gradual binding of IL‐25 to these two subunits in order to mediate downstream signaling cascades. In contrast to IL‐17, which induces Th1‐prone inflammation, IL‐25 is implicated in a Th2 response by enhancing the production of Th2 transcription factors through an IL‐4‐independent mechanism and directly activating Th2 memory cells to generate Type 2 cytokines [32]. The induction of Type 2 inflammation by IL‐25 leads to increased levels of serum IgE, blood eosinophils, and mucus production in asthmatic patients [33, 34]. IL‐25 also promotes airway remodeling in asthmatic patients. In the airways of mice induced by IL‐25, there is an increased population of fibroblasts expressing IL‐17RB [35]. Additionally, IL‐25 can induce fibroblast differentiation into myofibroblasts and subsequently enhance the deposition of extracellular matrix components such as Type I/III collagen and fibronectin in the lungs [36]. Moreover, IL‐25 induces heightened epithelial damage, sustains the infiltration of various immune cells in the airway, and establishes a positive feedback pathway, all of which contribute to facilitating airway remodeling [37]. The confirmation of the efficacy and safety of anti‐IL‐25 antibody biologics in clinical trials is still pending.

### 2.2. Airway Smooth Muscle Cell (ASMC) Proliferation and Airway Remodeling

The ASMCs originate from mesenchymal precursors and play a crucial role in the development of bronchial trees. ASMCs are believed to play a vital role in maintaining the basal airway tension and ensuring homogeneous lung ventilation by regulating local airflow resistance. The inflammatory stimulation of ASMCs induces AHR and leads to outcomes associated with airway remodeling [38]. Studies have shown that in asthmatic patients with Type 2 inflammation, eosinophils in the bloodstream can promote the proliferation of ASMCs and extracellular matrix, promoting airway remodeling [39]. The stimulation of IL‐13 in human ASMC induces the upregulation of RhoA protein, facilitating the release of calcium ion and subsequently leading to airway contraction [40]. In addition, Haruka et al. demonstrated that lymphotoxin *β* receptor (LT*β*R) induces smooth muscle remodeling and promotes the development of AHR in response to inhaled allergens through sustained activation of the NF‐*κ*B pathway [41].

In recent years, there has been an emerging trend in transcriptomic and microarray research. The aberrant expression of noncoding RNA (ncRNA) on ASMCs has been associated with asthma, with a specific emphasis on the exploration of microRNAs. The in vitro culture of ASMCs from allergic asthmatic patients has revealed that IgE downregulates the expression of phosphatase and tensin congeners (PTEN) by regulating microRNA‐21‐5p, thus enhancing the phosphatidylinositol 3‐kinase (PI3K) signaling. This ultimately promotes the proliferation of ASMCs and leads to airway remodeling [42]. MiR‐98‐5p downregulates the expression of RAS‐related C3 botulinum toxin substrate (RAC1), [43], and miR‐155‐5p inhibits the expression of TAB2 (TGF‐*β*‐activated kinase 1/MAP3K7‐binding protein 2) [44]. These two pathways can inhibit IL‐13‐induced ASMC proliferation and migration. These studies have proved the significant role of ASMCs′ ncRNA in the pathogenesis of Type 2 inflammation in asthma, thereby highlighting its promising research potential as a novel therapeutic target for severe asthma treatment.

## 3. Innate Immune Cells in Asthma With Type 2 Inflammation

### 3.1. ILC2 Possess a Robust Capability for Producing Type 2 Cytokines

The inherent lymphoid cells (ILCs) are derived from common lymphoid progenitor cells, belonging to the lymphoid lineage, and exhibit morphological similarities with T/B lymphocytes. However, unlike T/B lymphocytes, ILCs lack antigen recognition receptors and do not express lymphoid or myeloid markers [45]. ILCs, strategically positioned at barrier surfaces, serve as the primary immune responders against invading pathogens and may therefore determine the outcome of immune responses. Currently, distinct subsets of ILCs (ILC1, ILC2, and ILC3) have been characterized based on their phenotype and functionality. Notably, ILC2 predominantly resides in the airway and intestinal mucosa and is involved in Type 2 inflammatory responses of asthma [46].

The development of ILC2 and Th2 cells necessitates the involvement of a shared transcription factor, GATA3 [47]. Furthermore, the maturation and differentiation of ILC2 are linked with retinoid receptor‐related orphan receptor alpha (ROR alpha) and c‐MYC [48]. The recent elucidation reveals that the transcription factor RUNX1 is involved in activating ILC2 to produce Type 2 cytokines [49]. In OVA‐induced asthma models, studies have demonstrated that ILC2s contribute to the production of IL‐5 and IL‐13 in the lungs, comparable to Th2 cells, thereby inducing allergic airway inflammation [50]. This process mediates Type 2 airway inflammation. ILC2 has been demonstrated to autonomously produce Type 2 cytokines, independent of T cells [51]. The proliferative capacity of ILC2 and the secretion of IL‐5 and IL‐13 are enhanced in individuals with severe asthma. In murine model studies, the absence of PGE2 receptor EP2 resulted in an enhanced lung ILC2 response and endogenous eosinophilic inflammation induced by IL‐33. Mechanistically, PGE2 directly inhibits the activation of IL‐33‐dependent ILC2 through the EP2‐cAMP pathway, subsequently downregulating gene expression related to STAT5 and MYC pathways as well as energy metabolism in ILC2 [52]. Studies conducted on patients with eosinophilic asthma (EA) have found that IL‐33 can stimulate and activate ILC2 cells in the lungs via the PI3K/AKT pathway, resulting in enhanced production of Type 2 cytokines [28]. In addition to the typical epithelial alarmin IL‐33, the neuropeptide calcitonin gene‐related peptide (CGRP) was also identified in OVA‐induced mouse models as an activator of ILC2, driving Type 2 inflammation in asthma [53]. In a murine model of pulmonary innate Type 2 response (IT2IR), it is demonstrated that phospholipid scramblase‐1 (PLSCR1) interacts with chemoattractant receptor homologue 2 (CRTH2) to activate Group 2 innate lymphoid cells (ILC2s), which play a crucial role in the immune response associated with asthma Type 2 [54]. The confirmation of ILC2s′ ability to enhance eosinophil activity has been established. In turn, the complement C5aR1 of inflammatory eosinophils (iEOS) also has an effect on ILC2 activation; additionally, soluble mediators released by eosinophils induce proliferation of ILC2s [55, 56]. These findings unequivocally validate the existence of an interaction between eosinophils and ILC2s.

The resemblance in cytokine patterns generated by ILC2 and Th2 cells has prompted inquiries regarding the potential overlap of their functions in asthma. Katja has developed a novel mouse model that utilizes the neuromedium‐based U receptor 1 (Nmur1) promoter to simultaneously express Cre recombinase and green fluorescent protein, specifically targeting ILC2s while preserving the integrity of other immune cell populations. The nonredundant function of ILC2s in homeostasis and disease, in the presence of adaptive immune cells, has been validated [57], thereby promoting further investigations into the functionality of ILC2s. There is still a considerable distance to traverse in the exploration of functional disparities between Th2 cells and ILCs in the context of asthma.

### 3.2. Eosinophils Function as Effector Cells in the Immune Response

Eosinophils in the bone marrow originate from CD34+ hematopoietic stem cells, and their maturation is regulated by cytokines, primarily granulocyte and macrophage colony‐stimulating factor (GM‐CSF), interleukin‐3 (IL‐3), and IL‐5. Among them, IL‐5 is the most important, which exerts a profound impact not only on the maturation of eosinophils in the bone marrow but also on the initiation of their release into the peripheral circulation and extension of their lifespan [58]. The activation of eosinophils plays a pivotal role in the Type 2 inflammatory cascade of asthma, functioning as effector cells by releasing granular mediators and producing cytokines. Eosinophils adhere to endothelial cells by means of the interaction of glycoprotein PSGL‐1, vascular endothelial cell adhesion molecule 1 (VCAM‐1), and various integrins, thus facilitating their migration into peripheral tissues for the release of cytotoxic proteins (EPO, ECP, MBP, EPX, and EDN) and degranulation. This process triggers Type 2 inflammation and subsequently leads to the manifestation of asthma symptoms. Repetitive eosinophilic inflammation can also induce damage to structural cells within the airways, ultimately leading to airway remodeling.

Eosinophils are capable of forming eosinophilic extracellular traps (EETs) in response to various stimuli, which confine pathogens within a controlled range to safeguard the body. However, research has demonstrated that excessive production of EETs during chronic inflammation can exacerbate the inflammatory response, and the severity of the disease is closely associated with levels of EETs [59–61]. The formation of EETs is correlated with Charcot–Leyden crystals (CLCs), which are derived from galectin‐10 (Gal‐10) [62]. The binding of Gal‐10 to OVA induces a Type 2 immune response, resulting in an increase in airway eosinophils and IgE [63]. It has been reported that glutathione (GSH), which targets CLCs, disrupts Gal‐10 CLC formation and inhibits the crystallization of human Gal‐10 by chemically modifying Cys57, thereby ameliorating asthma symptoms [64].

The implementation of flow cytometry, a cutting‐edge technology, enables more precise classification of the functional status or subgroups of eosinophils. Based on the expression of CD101, eosinophils are divided into resident eosinophils (rEOS) that do not express CD101 and iEOS that express CD101. iEOS are recruited into the airway during the development of allergic airways [65]. According to the expression of CD62L, iEOS were further classified into distinct subgroups, among which CD62L^low^iEOS exhibited a significant association with asthma. A notable elevation in circulating CD62L^low^iEOS cells was observed in patients with severe EA, particularly those with comorbid nasal polyps. Nasal polyp tissues displayed preferential accumulation of CD62L^low^iEOS [66]. After mepolizumab treatment (a monoclonal antibody targeting IL‐5), there was a significant decrease in CD62L^low^iEos levels among patients with severe asthma [67]. Accurate classification of asthma holds potential for enhancing the efficacy of personalized asthma treatment.

### 3.3. DCs—The Role of Antigen Presentation

DCs predominantly originate from common dendritic cell progenitor cells (CDPs) in the bone marrow, with a subset derived from monocytes. The DCs′ subtypes include plasmacytoid dendritic cells (pDCs) and myeloid dendritic cells (mDCs, also known as conventional dendritic cells or cDCs). Myeloid DCs can be further classified into cDC1 and cDC2 subsets, while the monocyte‐derived subtypes are referred to as mocDCs [68]. DCs, which bridge the gap between innate and adaptive immunity, play a pivotal role in initiating and sustaining Type 2 inflammation in asthma. Among these subsets, the cDC2 subgroup has been extensively investigated and demonstrated to be indispensable for differentiating Th2 cells within the pulmonary microenvironment [69–71]. The recent studies have provided insights into the crucial role of cDCs (CD1C‐expressing DC2 cells) in maintaining Type 2 inflammation and facilitating the upregulation of genes associated with pathological airway remodeling [72]. DCs serve as a highly efficient antigen‐presenting cell (APC), exhibiting a diverse array of PRRs on their cell surface, including toll‐like receptors, C‐lectins, and cytoplasmic sensors. These PRRs recognize pathogen‐associated molecular patterns (PAMPs) and DAMPs, thereby activating and maturing DCs. The subsequent process involves the presentation of antigenic peptides by MHCII, along with the expression of costimulatory molecule B7 and OX‐40 ligand on DCs. CD4+ T cells are induced to differentiate into Th2 cells which produce Type 2 cytokines and eosinophilic inflammation. Ultimately, this cascade leads to a Type 2 immune response [73]. In addition to inducing a Type 2 response through the stimulation of DC maturation, epithelial‐derived cytokines (IL‐33, IL‐25, and TSLP) can also directly augment the expression of OX‐40 ligand on the surface of DCs, thereby promoting Th2 cell functionality and facilitating the progression of allergic asthma [74].

The number of cDC2s is elevated in patients with allergic asthma, and they respond by effectively binding to IgE through the expression of the high‐affinity receptor Fc*ε*RI, the ligand CD86 and OX‐40 [75, 76]. DCs can also migrate to lymph nodes, facilitating Th2 cell polarization, a process reliant on the interaction between CCR7 and its ligand CCL21. Additionally, recent findings have demonstrated that another ligand of CCR7, namely, CCL19, induces Th2 polarization in allergic airway diseases [77]. Moreover, CD109, a glycosylphosphatidylinositol anchored glycoprotein expressed in cDC2s, is implicated in the pathogenesis of asthma. CD109‐deficient mice exhibit reduced AHR, eosinophilic inflammation, and decreased expression of Th2 cytokines [78]. However, the specific underlying mechanism remains unexplored.

Recent studies have focused on targeting DCs as a potential strategy for mitigating Type 2 inflammation in asthma. The administration of CD200Fc in a murine model of HDM‐induced asthma resulted in the activation of the anti‐inflammatory pathway mediated by CD200/CD200R, leading to a reduction in cDC2 activation and disruption of the inflammatory cascade involved in the pathogenesis of chronic allergic asthma [79]. Miller et al. [3] screened the HDM receptor LMAN1 and revealed its expression in both DCs and airway epithelial cells. Furthermore, she demonstrated its potential to mitigate the inflammatory response by effectively downregulating NF‐*κ*B signaling through allergen binding [80]. Moreover, C1q is selectively enriched in lung CSF1R + cDC2s, and the depletion of C1q significantly attenuates allergen perception and asthmatic characteristics [81]. The role of DCs in the pathogenesis of Type 2 inflammation–associated asthma is under constant investigation.

### 3.4. Innate‐Like T (ILT) Cells—A Distinct Subset of Innate Immune Cells

ILT cells are a unique subset of unconventional T lymphocytes, primarily composed of invariant natural killer T (iNKT) cells, mucosal‐associated invariant T (MAIT) cells, and *γδ*T cells. These ILTs secrete various cytokines, including IL‐4, IL‐13, and IL‐17, in a manner dependent or independent of the T cell receptor (TCR). The TCR expressed by ILTs exhibits restricted and conservative characteristics [82]. The subtypes of ILT are classified based on distinct cytokine profiles. This review mainly elucidates the subtypes associated with Type 2 inflammation.

#### 3.4.1. iNKT Cells—Activated by Specific Recognition of Lipid Antigens

The iNKT cells undergo differentiation and maturation within the thymus, while also serving as “sentinels” in various tissues including the lung and liver. iNKT cells are activated rapidly by binding to lipid antigens presented by CD1d molecules, with alpha galactosylceramide (*α*‐GalCer) being particularly effective [83–85]. In allergic asthma, iNKT cells can evade apoptosis by upregulating the expression of acetyl‐CoA carboxylase 1 (ACC1), a key enzyme involved in de novo fatty acid synthesis [86], thereby ensuring their sustained participation in the pathogenesis of asthma. The iNKT2 subgroup, characterized by the secretion of IL‐4 and IL‐13, is predominantly associated with Type 2 inflammation. The development of iNKT2 cells relies on the transcription factors promyelocytic leukemia zinc finger (PLZF), GATA3, and IRF4 [87–89]. When lung tissue is attacked by antigens, iNKT cells are driven by chemokines such as TARC, CXCL9, and CXCL13 and chemokine receptors including CCR4, CCR9, and CXCR6. Subsequently, iNKT cells migrate into the lungs to exert their functional role [90, 91]. Notably, CCR4 plays a key role in mediating the migration of iNKT cells. Depletion of CCR4 impedes the migration of iNKT cells toward lung tissue, thereby ameliorating antigen‐ or *α*‐GalCer‐induced AHR in mice [92]. In addition, significantly elevated TARC was also observed in BALF and induced sputum collected from asthmatic patients [93, 94]. After antigen recognition, iNKT cells are activated within the lung tissue. On the one hand, iNKT cells themselves have the capability to secrete IL‐4 and IL‐13 [95]. On the other hand, they can also modulate the activity of other cell types such as macrophages and Th2 cells, thereby promoting increased secretion of IL‐4 and IL‐13 to initiate a Type 2 immune response [96, 97].

Previous studies in murine models have demonstrated iNKT cells can directly induce asthma [98], and our ultimate objective is to regulate iNKT cells′ activation for alleviating asthma. The iNKT cells exhibit specific recognition toward lipid antigens, while ɑ‐Lancer functions as an analog of ɑ‐GalCer to competitively inhibit the activation of iNKT cells [99]. The administration of anti‐CD1D resulted in diminishing the abundance and activation of pulmonary iNKT cells in asthmatic mice, thereby inhibiting the Th2 cell response through the suppression of immunogenic maturation of DCs that is dependent on pulmonary iNKT cells [100]. EZH2 has been demonstrated to possess the capacity to impede iNKT cell proliferation and decrease IL‐4, IL‐13, and IgE [101]. Furthermore, EZH2 induces downregulation of prostacyclin (PGRN) signaling, which stimulates iNKT cells to produce IL‐4 and IL‐13 [102]. Thus, the Th2 response induced by iNKT cells can be effectively regulated through the PGRN‐EZH2‐PLZF pathways. Intriguingly, Li et al. [103] discovered that treatment with oral antibiotics can reduce iNKT cells and alleviate allergic asthma symptoms.

#### 3.4.2. *γδ*T Cells—A Less Explored Cellular Subtype

The proportion of *γδ*T cells is relatively small; however, they have a certain impact on the pathogenesis of asthma, as evidenced by an observed elevation in *γδ*T cell levels among asthmatic patients [104]. The TCR expressed by *γδ*T cells differs from that of other T cell types in its composition, which includes gamma and delta chains. It is also independent of the major histocompatibility complex (MHC) and exhibits rapid responsiveness to pathogens [105]. The subsets of *γδ*T cells capable of producing IL‐4 and IL‐13 were categorized as Th2‐like *γδ*T cells [106]. Mice and humans exhibit distinct populations of *γδ*T cells within the respiratory system, in which the two subtypes of mice, V*γ*1*δ*1T cells and V*γ*4*δ*1 T cells, exert contrasting effects by promoting and suppressing allergic reactions, respectively [107, 108]. Similar findings have been reported in studies of human asthma, with some studies observing an upregulation of *γδ*T cells [109] while others suggesting no association between *γδ*T cells and asthma or even a reduction in their frequency [110]. However, when considering specific subtypes, a consistent conclusion can be drawn that patients with allergic asthma exhibit an increased presence of peripheral blood *γδ*T cells producing Type 2 cytokines [111, 112]. Limited research has been conducted on the role of *γδ*T cells in asthma; however, it is evident that distinct cell subpopulations exist among different phenotypes of asthma patients. Therefore, future investigations should focus on studying these diverse subpopulations of *γδ*T cells to enhance our comprehension.

#### 3.4.3. MAIT Cells—Regulatory Potential in Asthma

Human lung tissue harbors a substantial population of MAIT cells, which recognize antigens presented by major MHCI‐related protein 1 (MR1) in mammals. These MAIT cells can be further classified into two subtypes, namely, MAIT1 and MAIT17, based on their expressed transcription factors and secreted cytokines [113]. Specifically, the MAIT1 subset produces IFN‐*γ*, while the MAIT17 subset secretes IL‐17A [114, 115]. In studies investigating the correlation between MAIT cells and human asthma, researchers observed a significant decrease of MAIT cells in peripheral blood, sputum, BALF, and lung tissues of T2‐high asthma compared to healthy controls [116]. Furthermore, there was also a lower frequency of circulating MAIT cells found in asthma patients who responded to mepolizumab (anti‐IL‐5mab) [117]. These findings suggest that MAIT cells possess the potential to alleviate Type 2 inflammation. In a murine model of allergic airway inflammation, it was observed that MAIT cells can suppress ILC2 proliferation and induce the production of Type 2 cytokines through IFN‐*γ* secretion by MAIT1 [118, 119].

### 3.5. MCs—Exclusively Associated With IgE in the Context of Asthma?

MCs are highly granular tissue–resident cells derived from bone marrow hematopoietic stem cells and localized in tissues in close proximity to the external environment, such as lung mucosa and skin [120]. They respond to infection and harmful stimulation by releasing various mediators after being activated by binding with IgE.

MC degranulation is a hallmark of allergy. In the context of asthma, it has been extensively investigated that MCs are activated through the binding of high‐affinity IgE receptor Fc*ε*RI on their surface to allergen–IgE complexes, thereby triggering the release of degranulation mediators (such as histamine, prostaglandin, leukotrienes, 5‐hydroxytryptamine, chemokines, and IL‐13), ultimately leading to the allergic asthma symptoms. In addition to its close relationship with IgE, IL‐33 activates and proliferates MCs by binding to ST2 receptors on MCs, subsequently leading to the release of Type 2 cytokines [121]. IL‐33 can also enhance the production of CysLT, which is produced by eosinophils and interacts with MCs to induce Type 2 inflammation [122, 123]. However, a study conducted by Angelica et al. [124] revealed that IL‐33‐induced MC signaling was associated with severe neutrophilic asthma, while IgE‐mediated MC activation was linked to EA. This distinction may be attributed to the remission of eosinophilic proliferation in these severe asthmatic patients following glucocorticoid treatment.

Inhibiting the proliferation and activation of MCs can alleviate asthma symptoms driven by Type 2 inflammation, and corticosteroids currently serve as the primary treatment for asthma related to Type 2 inflammation. However, significant progress has been achieved in recent years in elucidating the underlying mechanism responsible for the therapeutic efficacy of natural compounds in treating asthma. Ingredients in solasodine [125], a natural medicine from India, and the herb *Acalypha indica* Linn [126] inhibit MC degranulation and effectively reduce the production of histamine and Type 2 cytokines in the airway. Kaurenoic acid derived from the Brazilian traditional drug Pruski can effectively reduce lung MC infiltration in OVA‐induced mouse asthmatic models, suppressing the Th2 pathway and decreasing levels of IL‐33, IL‐4, and IL‐5 in BALF [127]. A variety of Chinese herbal extracts mugwort [128], ephedra [129], and calamus [130] extracts have been demonstrated to attenuate MC infiltration in the lungs of asthmatic mouse models. Moreover, these extracts have exhibited the ability to downregulate Type 2 cytokines and ameliorate asthma symptoms.

### 3.6. Basophils—Similar to MCs

Basophils, being the least abundant circulating granulocytes, share developmental origins with MCs. They exhibit certain characteristics similar to MCs and play a pivotal role in allergic inflammation. High‐affinity IgE receptors are expressed on the surface of basophils, which, upon crosslinking with IgE, become activated and subsequently release histamine, Type 2 cytokines, and other proinflammatory mediators. This leads to an increase in vascular permeability and infiltration of eosinophils. Studies have shown that within the context of allergic inflammation, IL‐33 has been proven to rapidly induce the secretion of IL‐4 and IL‐13 by basophils, surpassing the secretion levels observed in Th2 cells and MCs, while TSLP does not exhibit this effect [131, 132]. Basophils can also modulate the infiltration of eosinophils [133]. By studying the quantitative PCR expression levels of genes in sputum of patients with severe asthma, Natasha found that these basophil‐related genes (GATA2, TPSB2, CPA3, GPR56, HDC, and SOCS2) were correlated with eosinophilic airway inflammation [134]. Further investigation into the specific mechanisms by which basophils regulate eosinophils, as well as the distinctions between basophils and MCs, may provide a more profound comprehension of asthma driven by Type 2 inflammation.

## 4. Adaptive Immune Cells in Asthma With Type 2 Inflammation

### 4.1. T Helper (Th) Cells in Pathogenesis of Asthma

Th cells originate from pluripotent stem cells in the bone marrow. Th cells constitute a distinct subset of T cells, and under the guidance of specific transcription factors, naive Th cells undergo differentiation into various subgroups. Th2, TFH, and Th9 are mainly associated with asthma driven by Type 2 inflammation.

#### 4.1.1. Activation of the Th2 Pathway Lies at the Core of Type 2 Inflammation

The differentiation of naive Th cells into Th2 cells is predominantly regulated by the transcription factor GATA3. In contrast to the innate immunity mediated by ILC2s, Th2 cells modulate adaptive immunity that secretes copious amounts of IL‐4, IL‐5, and IL‐13 in an antigen‐dependent manner [135], thereby playing a pivotal role in Type 2 inflammation in asthma.

IL‐4 and IL‐13, which share the Type 2 receptor IL‐4R, play a crucial role in the pathogenesis of airway hyperresponsiveness, goblet cell metaplasia, and increased production of airway mucus in patients suffering from allergic asthma. These two cytokines also upregulated the expression of VCAM‐1 on endothelial cells, facilitating the recruitment of eosinophils and other inflammatory cells to the site of inflammation [136]. IL‐4 upregulates the Th2 cell transcription factor GATA3 in naive Th cells, thereby sustaining the proliferation of Th2 cells and facilitating the proliferation of B cells as well as the production of IgE [137]. Meanwhile, IL‐13 is responsible for the airway remodeling, as well as the production of IgE [138]. IL‐5 plays a key role in the maturation, differentiation, release, and recruitment of eosinophils, while also exerting influence on eosinophils′ survival. Currently, the FDA has approved biologics targeting typical Type 2 cytokines and their receptors (such as the anti‐IL‐5 monoclonal antibody mepolizumab, anti‐IL‐13 monoclonal antibody lebrikizumab, and anti‐IL‐4R monoclonal antibody dupilumab) for clinical use in treating severe asthma driven by Type 2 inflammation. These biologics demonstrate significant efficacy in controlling asthma symptoms, reducing eosinophils and fractional exhaled nitric oxide (FeNO), improving forced expiratory volume in 1 s (FEV_1_), and enhancing overall quality of life for individuals with asthma.

#### 4.1.2. Follicular T Helper (TFH) Cells Are Likely to Be Crucial in Regulating the IgE Class Switching

Naive CD4+ T cells undergo differentiation into TFH cells primarily under the influence of the transcription factor Bcl6, and accumulating evidence suggests that TFH cells, rather than Th2 cells, predominantly regulate IgE antibody class switching in asthma [135]. The subtypes responsible for IgE production and Type 2 cytokine secretion, including TFH2 and TFH13 cells, play a crucial role in the pathogenesis of allergic asthma [139]. The findings of a study conducted on children with allergic asthma showed a positive correlation between the frequency of TFH13 cells and dust mite–specific IgE, suggesting that the TFH13 cells may be responsible for the immunopathological mechanism leading to excessive IgE accumulation in pediatric patients with allergic asthma [140]. The maintenance of TFH phenotype following T cell differentiation is uniquely reliant on continuous ICOS signaling, and blocking ICOS signaling effectively depletes TFH cells while reducing allergen‐specific IgE levels, pulmonary IL‐13 expression, and AHR [141]. The proportion and count of circulating TFH2 and ICOS + cTFH cells exhibited a decline in patients with allergic asthma who underwent treatment with ICS and an anti‐IgE mAb (omalizumab) [142–144]. These researches highlight the indispensable role of TFH cells in asthma with Type 2 inflammation.

#### 4.1.3. Th9 Cells May Have Been Underestimated in Allergic Asthma

Th9 cells are induced during the activation of naive Th cells in the presence of both IL‐4 and TGF‐*β* [145]. The transcription factors STAT6, IRF4, and GATA3 play essential roles in the development of Th9 cells. Additionally, BATF, NF‐*κ*B, and STAT5 also contribute to the polarization of Th9 cells [146]. A novel transcription factor, Id1, has been identified to play a crucial role in Th9 cell differentiation by inhibiting Tcf3 and Tcf4, which are capable of binding to the IL‐9 gene and thereby suppressing its expression [147]. Th9 cells are a unique subset of Th cells, primarily characterized by their production of the cytokine IL‐9. IL‐9 promotes the proliferation of Type 2 cells (including Th2 cells and ILC2 cells) as well as facilitating the generation of Type 2 cytokines. Additionally, IL‐9 is involved in MCs′ proliferation and migration, which serves as a pivotal factor in the pathogenesis of allergic asthma. In a murine asthmatic model, dual specificity phosphatase 8 (DUSP8) mediates Th9‐induced allergic response by impeding the nuclear translocation of transcriptional repressor Pur‐ɑ [148]. The expression of IL‐9 was also found to be elevated in peripheral blood mononuclear cells (PBMCs) derived from patients with allergic asthma [149]. IL‐9 has been overlooked due to its classification as a Th2 cytokine. So far, no specific transcription factor has been identified in association with Th9 cells. Further investigation is warranted to elucidate the role of Th9 cells in asthma.

### 4.2. B Cells Act Through Producing Antibodies and Cytokines

Memory B cells produce IgE, a specific hallmark of Type 2 immunity, which is an important research area in the context of Type 2 inflammation of asthma. The high‐affinity IgE receptor FcɛRI is expressed on both immune and structural cells encompassing basophils, MCs, eosinophils, epithelial cells, and airway smooth muscle. Upon binding to FcɛRI, IgE triggers activation of these cells leading to the initiation and progression of asthma. Recent studies have shown that the control of IgE class switching in asthma is closely associated with TFH cells producing IL‐4 and IL‐13 [135].

In addition to their involvement in Type 2 inflammation by producing IgE, B cells also contribute to asthma driven by Type 2 inflammation through the secretion of other antibodies. IgA, the predominant immunoglobulin in the mucosa, has garnered increasing attention for its role in asthma. In an asthmatic murine, the levels of IgA were increased in both serum and BALF [150]. In asthmatic patients, the number of IgA+ memory B cells in the bloodstream is increased. However, whether IgA+ memory B cells are associated with Type 2 inflammation in asthma has not been further investigated [151]. These studies all indicate the potential involvement of IgA in the mucosal pathogenesis of asthma. IgD, another antibody produced by B cells, exhibits an opposite effect in airway diseases. The number of IgD+ memory B cells increased significantly during specific immunotherapy in asthmatic patients sensitized to house dust mites [152], indicating that IgD might induce immune tolerance. However, in patients with chronic rhinosinusitis with nasal polyps (CRSwNP), IgD has been found to stimulate MCs for the production of IgE and promote Type 2 inflammation [153]. Furthermore, it has been observed that IgD can also augment the secretion of IL‐4, IL‐13, and IL‐5 by basophils and TFH cells upon antigenic stimulation [154]. Elevated IgD+ IgM‐memory B cells have been observed in asthmatic patients with variant immunodeficiency; however, further validation is required in nonimmunodeficient individuals with asthma. The role of IgD in asthma needs further investigation.

In addition to producing diverse antibodies involved in inflammation, B cells also contribute to immune reaction by producing the cytokine IL‐10. FoxO1, an upregulated inhibitory factor in mice with allergic asthma, is believed to exert suppressive effects on the expression of transcription factors, which induce the production of IL‐10 by B cells [155]. However, recent studies have revealed that B cell‐derived IL‐10 exhibits an allergenic effect, while Bcl‐3 exerts a preventive role against HDM‐induced asthma by suppressing the production of IL‐10 by B cells [156]. The biological function of B cells is intricate, necessitating further investigations in both asthma models and clinical cohorts to elucidate their role in this disease.

## 5. Conclusion

Asthma is considered to be a typical “Type 2 disease.” Owing to the substantial prevalence of Type 2 inflammation in asthma patients, an increasing number of studies have been conducted on Type 2 inflammation in asthma. The heterogeneity of asthma is directly associated with the diverse array of immune cells and structural cells implicated in the pathogenesis of asthma, thereby posing a significant challenge to achieving optimal clinical outcomes. This review synthesizes multiple cell types involved in the inflammatory cascade underlying Type 2 asthma, aiming to provide invaluable insights into the investigation of the mechanism of biologics. Despite significant advancements in the study of Type 2 inflammatory pathways in asthma, further research is urgently needed in this field.

## Conflicts of Interest

The authors declare no conflicts of interest.

## Funding

This study was supported by the State Key Laboratory of Respiratory Disease, 10.13039/100013262 (SKLRD‐L‐202603).

## Data Availability

Data sharing is not applicable to this article as no datasets were generated or analyzed during the current study.
